# Outcomes of a third course of salvage spine stereotactic radiosurgery for spinal metastases: a single-institution case series

**DOI:** 10.1007/s11060-026-05581-9

**Published:** 2026-04-22

**Authors:** Jessica J. Bai, Ehsan H. Balagamwala, Anthony Magnelli, Lilyana Angelov, John H. Suh, Erin S. Murphy, Praveen Pendyala, Samuel T. Chao

**Affiliations:** 1 Case Western Reserve University School of Medicine, Cleveland, OH USA; 2 Department of Radiation Oncology, Cleveland Clinic Foundation, Cleveland, OH USA; 3 Department of Neurological Surgery, Cleveland Clinic Foundation, Cleveland, OH USA; 4 Rose Ella Burkhardt Brain Tumor and Neuro-oncology Center, Cleveland Clinic Foundation, Cleveland, OH USA; 5 Cleveland Clinic Lerner College of Medicine of Case Western Reserve University, Cleveland Clinic Taussig Cancer Center, 10201 Carnegie Ave, Cleveland, OH 44106 USA

**Keywords:** Stereotactic radiosurgery, Metastases, Central nervous system

## Abstract

**Objective:**

Re-irradiation with salvage spine stereotactic radiosurgery (sSRS) has emerged as a viable strategy for spinal metastases with progressive disease. We report the characteristics and clinical outcomes of spinal segments that received at least three courses of sSRS.

**Methods:**

10 spinal segments in 6 patients who received at least three courses of sSRS were evaluated from an Institutional Review Board-approved retrospective single-institution database. Overall survival (OS) and radiographic progression-free survival (rPFS) were calculated by Kaplan-Meier analysis. Radiographic failure was defined as progression on imaging at the treated segment.

**Results:**

Median follow-up was 8.9 months (range, 0.2–46.3). Three patients had died at the time of analysis. Median age at treatment was 63.2 years (range, 36.9–77.2), and median Karnofsky Performance Status (KPS) was 80 (range, 70–90). The 1-year overall survival (OS) rate was 50.0%. The median cumulative thecal sac EQD2_2.5_ D_max_ after the third course of sSRS was 137.9 Gy (range, 97–227.5). The median third course re-irradiation sSRS thecal sac EQD2_2.5_ D_max_ was 37.15 Gy (range, 21.8–57.8). 30% (3/10) of treated segments had radiographic progression, with a 6-month rPFS rate of 85.7%. 28.6% (2/7) of treatment courses resulted in pain flare. No cases of vertebral compression fracture (VCF), radiation myelopathy, or neuritis were reported.

**Conclusions:**

Three courses of sSRS may be considered for salvage treatment of progressive spinal metastases. Given that cumulative neural structure doses exceed established thresholds, careful multidisciplinary review of all treatment options including surgery and other ablative treatments is essential before proceeding.

## Introduction

Spine stereotactic radiosurgery (sSRS) delivers high dose conformal radiotherapy in a few fractions, maximizing ablative tumor coverage while sparing adjacent organs at risk (OARs) such as the spinal cord and cauda equina [1, 2]. Re-irradiation with salvage spine stereotactic radiosurgery (sSRS) has emerged as a viable strategy for spinal metastases with progressive disease, yielding 1-year local control rates of ~ 80% and low rates of vertebral body fracture and radiation-induced myelopathy [3–5]. However, epidural tumor progression remains a risk in re-irradiation with sSRS, most likely due to conservative dosing of critical neural structures within the epidural space [1, 3, 6].

There is currently limited literature assessing multiple courses of sSRS after local tumor progression. We report the characteristics and clinical outcomes in patients who, after receiving re-irradiation with sSRS and experiencing subsequent radiographic progression, underwent a salvage third course of sSRS to the same spinal segment.

## Methods

Using an Institutional Review Board-approved single-institution database, we identified 10 spinal metastases in 6 patients who received at least three courses of sSRS to the same spinal segment. The primary objective was to determine radiographic failure rates after the salvage third sSRS course. Radiographic failure was defined as progression on imaging at the treated segment as determined by the multidisciplinary team including radiology, neurosurgery and radiation oncology. To assess toxicity outcomes, clinical notes and magnetic resonance images (MRI) were reviewed to determine pain flare, radiation-induced vertebral compression fracture (VCF), and radiation myelopathy. Pain flare was defined as an increase in pain within 1 week of treatment, requiring the initiation of steroids. Radiation-induced VCF was defined as fractures that occurred after radiation but before tumor progression, as tumor progression may independently cause destabilization [7]. Radiation myelopathy was defined as new onset or progressive neurological deficits attributable to radiation injury of the spinal cord, occurring in the absence of radiographic tumor progression at the treated level, and supported by MRI findings consistent with radiation-induced cord injury.

Our institution’s sSRS technique and setup has been previously described [8, 9]. The target volume was contoured to cover the entire vertebral body for lesions within the vertebral body [10]. For lesions affecting the lamina, pedicles, or spinous process, the target volume included all of the posterior elements. A planning target volume (PTV) margin was not utilized [10]. The spinal cord and thecal sac were contoured with 4.5 mm cranial and caudal margins. Treatment planning was performed using Pinnacle (Philips, Mayfield Heights, OH). sSRS was delivered using the Edge linear accelerator (Varian Medical Systems, Palo Alto, California, USA) equipped with 2.5 mm multi-leaf collimator leaves with cone beam CT (CBCT) image guidance. For patients treated with 5-fraction repeat sSRS, spinal cord was limited to < 0.35 cc receiving ≤ 22 Gy, with a maximum point dose capped at 28 Gy to mitigate the risk of myelitis. The cauda equina was constrained to < 5 cc receiving ≤ 30 Gy, with a maximum point dose of 31.5 Gy to reduce the risk of neuritis. For patients treated with 4-fraction repeat SRS, spinal cord was limited to < 0.35 cc receiving ≤ 18 Gy, with a maximum point dose limited to 25.6 Gy to reduce the risk of myelitis. Prophylactic corticosteroids were administered at the discretion of the treating physician, typically dexamethasone 4 mg daily tapered over two weeks or a methylprednisolone dose pack initiated one day prior to treatment, consistent with published protocols for pain flare prophylaxis in spine SBRT [11–13]. Pain flare was treated with dexamethasone 4 mg daily tapered over two weeks.

Clinical records, including consultation, completion, and follow-up notes, were systematically reviewed for each patient. Follow-up magnetic resonance imaging (MRI) of the treated spinal segment was performed at approximately 2–3 month intervals following each course of sSRS, or sooner if new or worsening neurological symptoms prompted earlier evaluation. Radiographic progression was determined by multidisciplinary consensus based on qualitative MRI review. Formal SPIne response assessment in Neuro-Oncology (SPINO) criteria were not prospectively applied given the retrospective design; future prospective studies in this setting should incorporate SPINO-based response assessment [14].

The treatment plans for each course of sSRS were reviewed for prescription dose, and the Dmax and D10% to the spinal cord and cauda equina for the salvage third course of sSRS were collected. Cumulative dose to the spinal cord and thecal sac was calculated using MIM software (MIM Software Inc., Cleveland, OH) by rigidly coregistering each treatment plan and accumulating dose across courses. The maximum point dose (Dmax), maximum dose covering 10% of the specified volume (D10%), and cumulative equivalent dose in 2 Gy fractions (EQD2₂.₅) Dmax were extracted from the dose-volume histogram of the accumulated dose distribution generated in MIM. The EQD2₂.₅ for each treatment course was calculated using the linear-quadratic model with an alpha/beta (α/β) ratio of 2.5 Gy, using the formula EQD2₂.₅ = D × (d + α/β) / (2 + α/β), where D is the total physical dose delivered in that course and d is the dose per fraction. All toxicity outcomes were graded per the Common Terminology Criteria for Adverse Events version 5.0 (CTCAE v5.0).

### Statistical analysis

Overall survival (OS), calculated from the start date of the salvage third sSRS course to the date of death or last follow-up, was estimated using the Kaplan-Meier method on a per-patient basis. Radiographic progression-free survival (rPFS), defined as the interval from the start date of the salvage third sSRS course to radiographic progression at the treated segment, death, or last follow-up, was estimated using the Kaplan-Meier method on a per-lesion basis. For patients with multiple segments treated simultaneously, each segment was analyzed independently for the purpose of rPFS. All statistical analyses were performed using R version 4.3.1 (R Foundation for Statistical Computing, Vienna, Austria).

## Results

10 spinal metastases in 6 patients were treated with a salvage third course of sSRS between January 2020 and December 2023 to the same spinal level after radiographic failure. Median time interval to sSRS re-irradiation was 12.1 months (range, 3.6–21.2). One of these segments subsequently received a fourth course of sSRS in December 2023. One patient received prior conventional external beam radiation therapy (EBRT) before their first course of sSRS; therefore, after their salvage third course of sSRS, they had undergone four radiation courses to the same metastatic spinal segment. 50% of patients were female, median age at treatment was 63.2 years (range, 36.9–77.2), and median consult KPS was 80 (range, 70–90). All patients had oligometastatic disease. Epidural disease was present in 60% (6/10) of treated spinal segments at the time of the third sSRS course. Of the 10 spinal segments treated, 50% (5/10) were treated primarily for pain palliation, 20% (2/10) for asymptomatic radiographic progression, and 30% (3/10) for combined pain and neurological deficit. Additional clinical characteristics can be found in Table 1.

**Table 1:** Clinical and treatment characteristics for spinal segments treated with a salvage third and fourth sSRS course

Characteristic	3 courses of sSRS		4 courses of sSRS	
	*n* = 10 spinal segments		*n* = 1 spinal segment	
Primary cancer				
Colorectal Adenocarcinoma	4	40.0%		1
Head & Neck NUT Midline Carcinoma	1	10.0%		-
Lung Carcinoid	1	10.0%		-
Renal Clear Cell	3	30.0%		-
Myxoid Liposarcoma	1	10.0%		-
Spinal level				
Thoracic	5	50.0%		-
Lumbar	1	10.0%		-
Sacral	4	40.0%		1
Reason for treatment				
Asymptomatic	2	20.0%		-
Pain	5	50.0%		-
Pain & Neurological deficit	3	30.0%		1
Spinal disease characteristics	6	60.0%		
Epidural Disease	6	60.0%		-
Paraspinal	6	60.0%		1
Vertebral Body	10	100.0%		1
Posterior Elements	2	20.0%		-
Single level	0	0.0%		-
Multilevel ≤ 5 VB	7	70.0%		-
Diffuse Spine Involvement	3	30.0%		1
Prior surgeries				
Prior cEBRT	1	10.0%		-
Prior hardware	3	30.0%		-
Prior kyphoplasty	1	10.0%		-
Salvage sSRS total prescription dose/number of fractions				
27 Gy/4 fractions	2	20.0%		-
27.5 Gy/5 fractions	1	10.0%		-
30 Gy/4 fractions	4	40.0%		1
30 Gy/5 fractions	3	30.0%		-

*Abbreviations*: sSRS = spine stereotactic radiosurgery; NUT = nuclear protein in testis; VB = vertebral body

The median dose for the first sSRS course was 30 Gy/3 fractions (range, 16–30 Gy/1–5 fractions). For the second sSRS course, the median dose was 30 Gy/4 fractions (range, 30 Gy/4 fractions – 30 Gy/5 fractions). Median dose for the third course of sSRS was 30 Gy/4 fractions (range, 27–30 Gy/4–5 fractions). Median cumulative Dmax and D10% to the spinal cord was 51 Gy (range, 50.1–71.1) and 45.8 Gy (range, 36.9–55.4), respectively. Median cumulative Dmax and D10% to the cauda equina was 99.5 Gy (range, 58.5–99.5) and 72.4 Gy (range, 34.9–72.4), respectively. A summary of the dosimetric factors can be found in Table 2. The median cumulative thecal sac EQD2_2.5_ D_max_ after the third course of sSRS was 137.9 Gy (range, 97–227.5). The median third course re-irradiation sSRS thecal sac EQD2_2.5_ D_max_ was 37.15 Gy (range, 21.8–57.8).

**Table 2:** Dosimetric factors for initial course sSRS, salvage second sSRS course, salvage third sSRS course for 10 spinal segments, and the salvage fourth sSRS course for 1 spinal segment

Factor	Initial sSRS course (*n* = 10)	Salvage second sSRS course (*n* = 10)	Salvage third sSRS course (*n* = 10)	Salvage fourth sSRS course (*n* = 1)
Median prescription absolute total dose in Gy/fractions with ranges	30 Gy/3 fractions (16–30 Gy/1–5 fractions)	30 Gy/4 fractions (30 Gy/4–5 fractions)	30 Gy/4 fractions (27–30 Gy/4–5 fractions)	30 Gy/4 fractions
Cord (*n* = 5)				
Median cumulative Dmax with range	10.4 Gy (9.8–23.13)	31.7 Gy (30.8–48.6)	51 Gy (50.1–71.1)	
Median cumulative D10% with range	8.9 Gy (8.19–18.9)	28.6 Gy (26.6–39.7)	45.8 Gy (36.9–55.4)	
Cauda Equina (*n* = 5)				
Median cumulative Dmax with range	19.5 Gy (17.8–19.5)	78.2 Gy (42.4–78.2)	99.5 Gy (58.5–99.5)	99.5 Gy
Median cumulative D10% with range	16.1 Gy (13.8–16.1)	50.6 Gy (34.3–50.6)	72.4 Gy (34.9–72.4)	72.4 Gy

*Abbreviations*: Dmax = maximum dose; D10% = maximum dose covering 10% of the specified volume

**Fig. 1:**
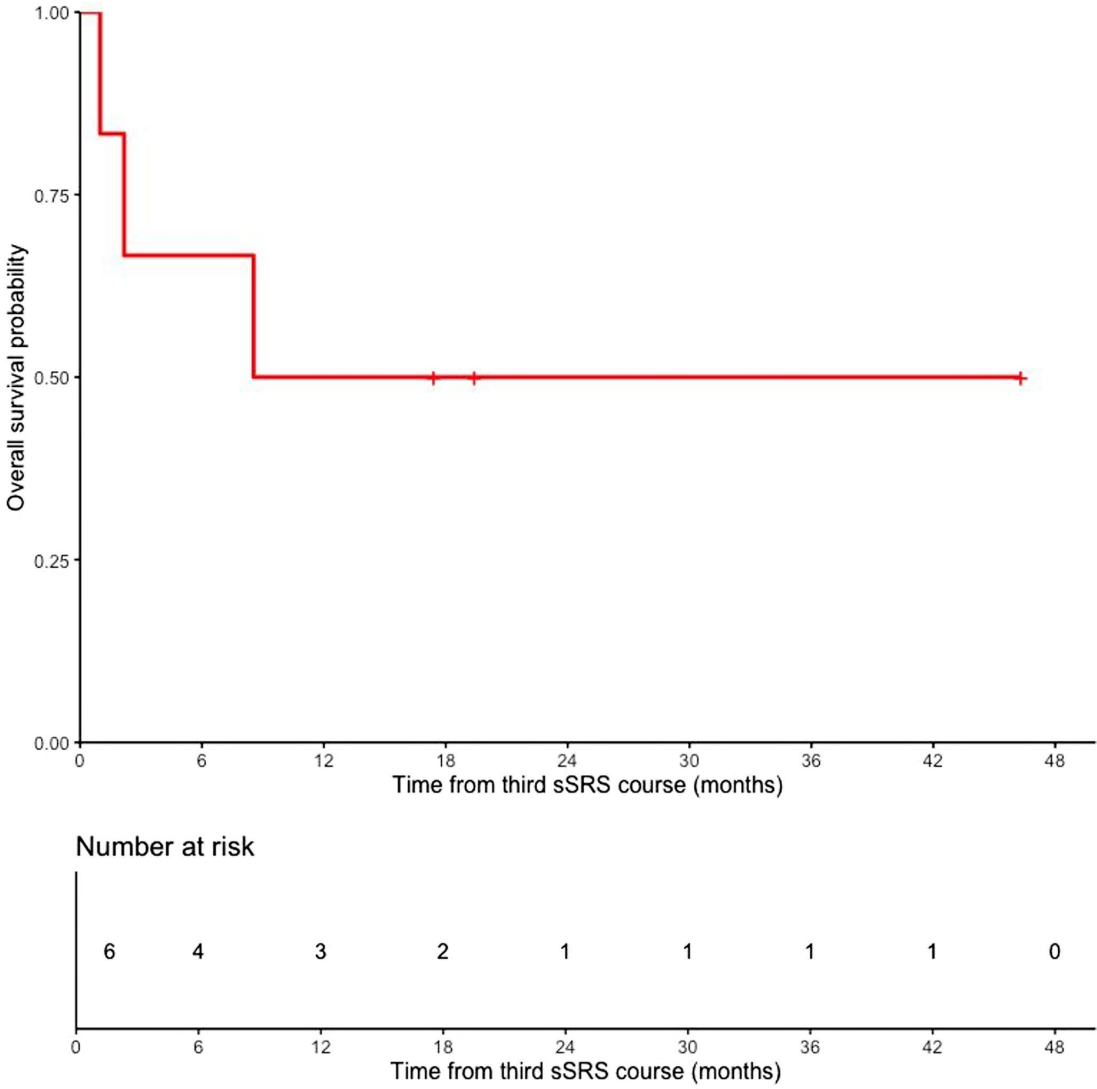
Overall survival after salvage third course spine stereotactic radiosurgery. The y-axis depicts the overall survival rate after salvage third course sSRS, and the x-axis depicts months from salvage third course sSRS. The median overall survival was 8.5 months (260 days; 95% CI: 2.2 months–not reached). The 1-month, 6-month, and 1-year survival rates were 83.3%, 66.7%, and 50.0% (95% CI for 1-year OS 22.5% – 100%), respectively

The median OS was 8.5 months (260 days; 95% CI 2.2 months – not reached) from the start of the salvage third sSRS course. At the time of analysis, 3 of 6 patients had died, with survival times of 1.0, 2.2, and 8.5 months from the third sSRS course. The median follow-up for surviving patients was 27.7 months (range, 17.4–46.3). The 1-month, 6-month, and 1-year survival rates were 83.3%, 66.7%, and 50.0% (95% CI for 1-year OS 22.5% – 100%), respectively (Fig. 1).

**Fig. 2:**
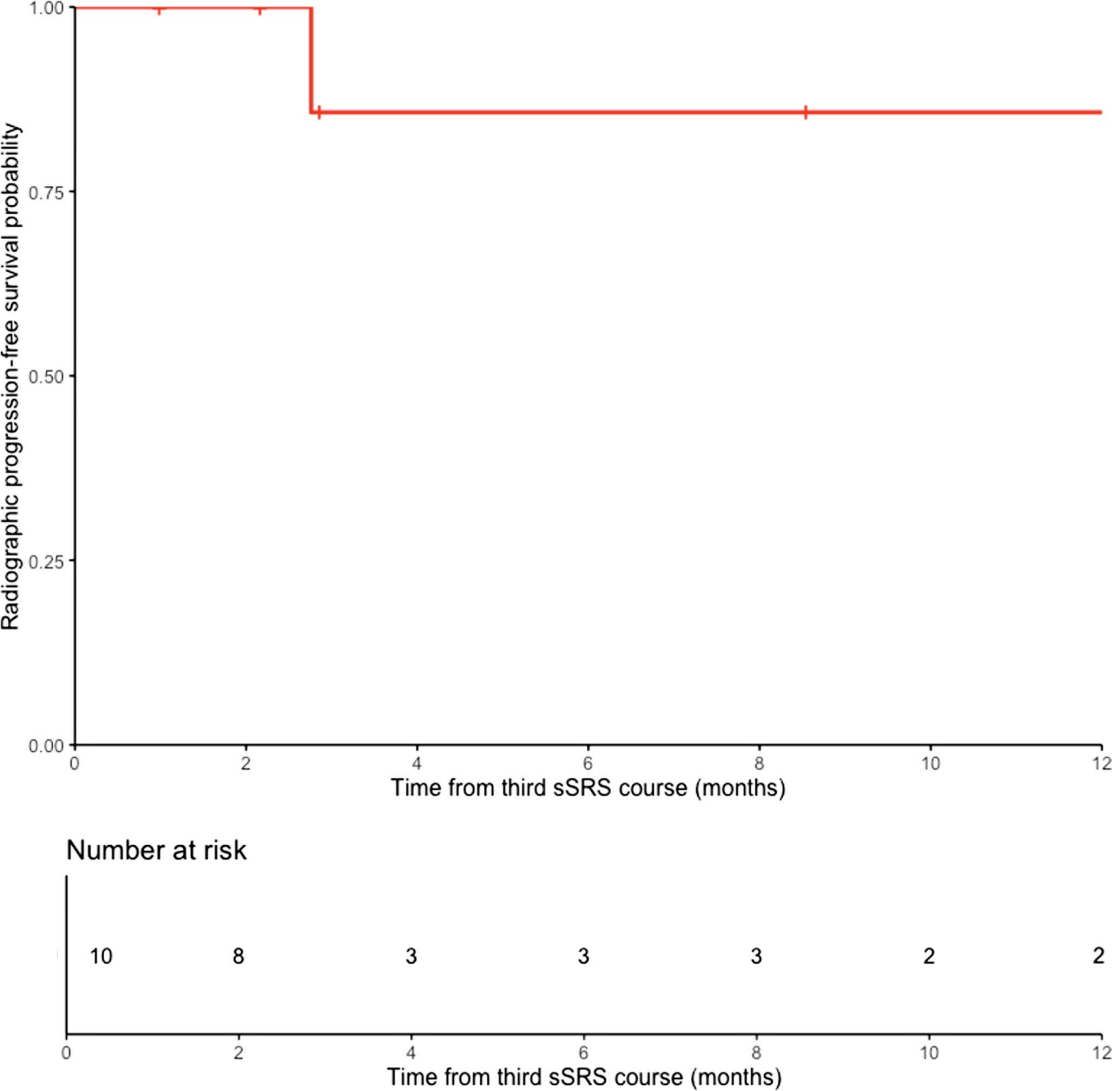
Radiographic progression-free survival (rPFS) rates after salvage third course spine stereotactic radiosurgery for the 10 spinal segments treated. The y-axis depicts the rPFS rate after salvage third course sSRS, and the x-axis depicts months from salvage third course sSRS. The 6-month rate of rPFS was 85.7% (95% CI 63.3% – 100%)

30% (3/10) of treated spinal segments had radiographic progression. The median time to radiographic progression was not reached at the time of analysis (range, 0.98–14.3 months). Median follow-up for lesions that did not experience radiographic progression was 2.8 months (range, 0.98–8.5) (Fig. 2). The 6-month rate of rPFS was 85.7% (95% CI 63.3% – 100%). Of the spinal segments that had radiographic progression, 33% showed radiographic progression within the paraspinal soft tissues, neural foramen, and pedicles, while 66% showed progression within the epidural space and vertebral body.

28.6% (2/7) of treatment courses resulted in pain flare, with onset between days 2 and 5 following treatment and resolution within 3 days in both cases with dexamethasone. Both cases were graded CTCAE Grade 2. Three patients received prophylactic corticosteroids prior to their third sSRS course, two with dexamethasone and one with methylprednisolone, and did not develop pain flare. No cases of VCF were reported after a salvage third course of sSRS. No cases of radiation myelopathy or neuritis were observed.

One patient who received 4 total courses of sSRS (4th sSRS dose 30 Gy/4 fractions) to the sacrum/coccyx is currently alive and at time of analysis, 99 days since the last course of sSRS, has not experienced radiographic failure. This patient subsequently developed left foot drop, which was attributed to repeated sacral irradiation and the proximity of the L5 nerve root to the treated field. This was further supported by MRI imaging demonstrating increased STIR signal in the left sacral nerve roots consistent with neuritis and EMG studies. The toxicity was graded CTCAE Grade 2, and the patient remains ambulatory with the assistance of an ankle-foot orthosis and a cane and has not become wheelchair dependent.

A detailed per-patient treatment timeline including histology, all prior courses of sSRS, prescription dose, systemic therapy at time of each course, systemic disease status, time interval from prior course, and cumulative thecal sac EQD2₂.₅ Dmax, pattern of progression, time to radiographic failure, vital status, and follow-up time from third course sSRS is provided in Table 3.

**Table 3:**
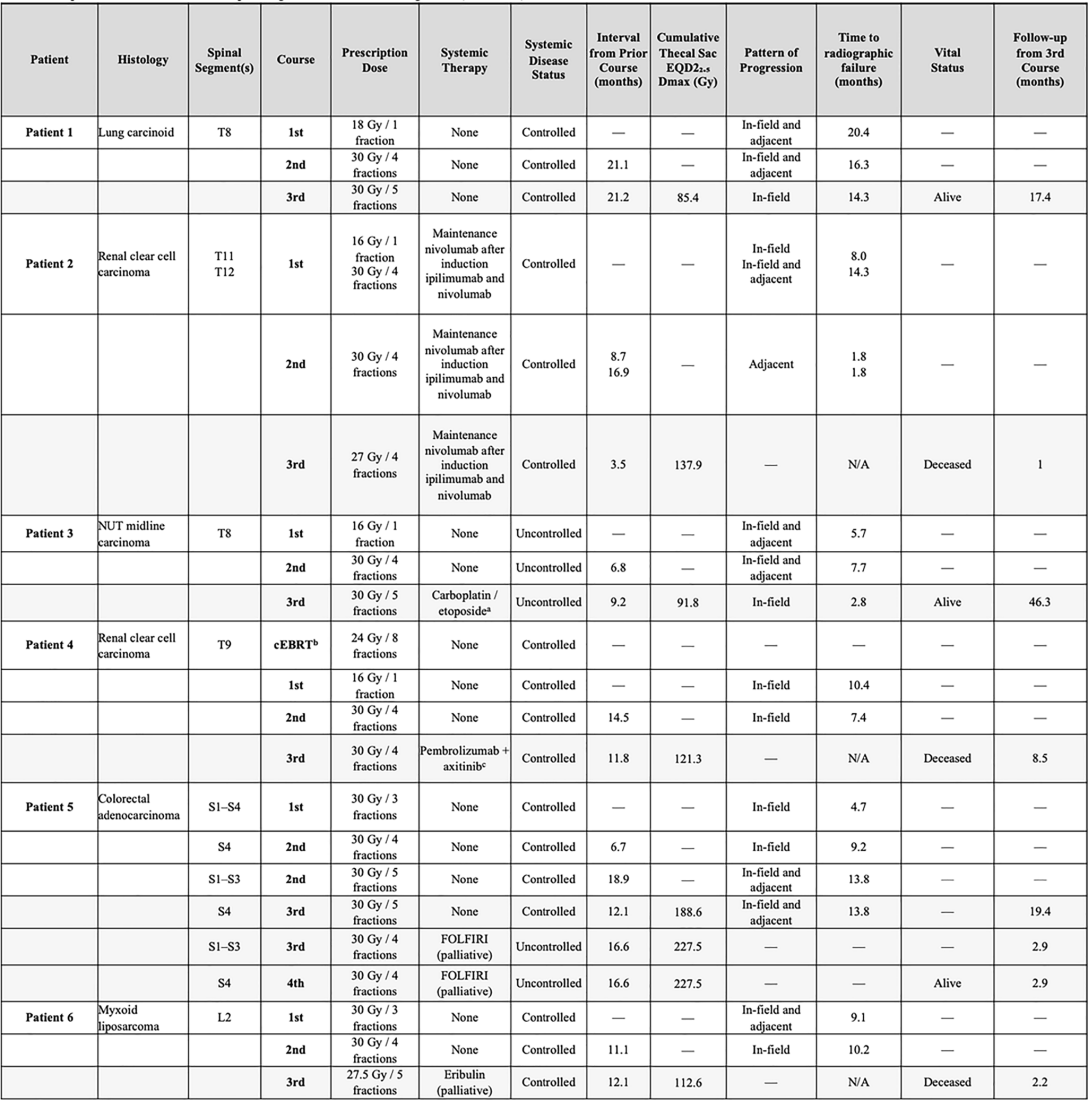
Per-patient treatment timeline for spinal segments treated with salvage third (and fourth) course sSRS

*Abbreviations*: sSRS = salvage spine stereotactic radiosurgery; EQD2₂.₅ = equivalent dose in 2 Gy fractions (α/β = 2.5 Gy); Gy = Gray; KPS = Karnofsky Performance Status; FOLFIRI = folinic acid, fluorouracil, irinotecan; Dmax = maximum dose; cEBRT = conventional external beam radiation therapy

^a^ Carboplatin/etoposide initiated 2 weeks following the third sSRS course

^b^ Patient had kyphoplasty followed by cEBRT to T9, received 24 Gy in 8 fractions (out of planned 30 Gy) in April of 2018, followed by laminectomy for cord compression 2 days later

^c^ Patient started pembrolizumab 2 days before and axitinib 5 days after radiation

## Discussion

This study represents one of the first efforts to investigate the efficacy and outcomes of multiple courses of salvage sSRS following local tumor progression. Our experience suggests that a salvage third course of sSRS is potentially safe and feasible in select cases, with goals including both symptom palliation and local radiographic control guided by individual clinical context.

Pain flare occurred in several patients with complete resolution with steroid use. Notably, three patients who received prophylactic corticosteroids did not develop pain flare, consistent with prior data demonstrating that prophylactic steroids reduce pain flare incidence following spine SBRT [11–13]. Pretreatment with steroids should be a consideration for salvage sSRS.

No instances of VCF, radiation myelopathy in cord-level segments, or radiation neuritis in cauda equina-level segments occurred, which is notable given the cumulative doses of radiation given. The absence of VCF may be partly attributed to prior surgical stabilization in several segments. 2/10 segments received hardware alone and 1/10 segments received both hardware and kyphoplasty. The two patients that received prior surgery had renal clear cell carcinoma as their primary, which has osteolytic properties known to increase fracture risk [15, 16]. Therefore, the added stability from hardware or kyphoplasty may have prevented VCF from occurring in these patients.

Our outcomes may suggest that salvage third course of sSRS is effective, as demonstrated by a favorable 6-month rPFS rate of 85.7%, though these results must be interpreted in the context of the 1-month, 6-month, and 1-year survival rates of 83.3%, 66.7%, and 50.0%, respectively. In one patient who received a fourth course of sSRS to the sacrum, this approach appeared feasible in the short term with no radiographic progression at 99 days; however, definitive conclusions cannot be drawn from a single case and further investigation is needed.

Epidural radiographic progression was one of the most common patterns of failure, which is consistent with prior studies [1, 3, 6]. This is attributable in part to the need to respect spinal cord and thecal sac dose constraints. Conventional EBRT may provide more uniform dose coverage of the epidural compartment, which can be advantageous in cases of extensive or circumferential epidural involvement. However, spinal cord tolerance remains critical regardless of modality, and treatment selection should be guided by disease geometry and the ability to safely achieve adequate target coverage. Paraspinal, neural foraminal, and pedicle progression occurred in 33.3% of progressing treatments, while 66.7% progressed within the epidural space and vertebral body, potentially reflecting conservative dosing and the inherent radioresistance of colorectal adenocarcinoma, lung carcinoid, and NUT midline carcinoma [17–19]. In patients with significant epidural disease, a multidisciplinary discussion weighing conventional EBRT, surgical decompression, and sSRS is essential. Treatment selection should be guided by goals of care. sSRS may be preferred when durable local control is desired, whereas conventional EBRT or palliative surgery may be more appropriate when rapid symptom relief is prioritized and cumulative cord dose limits preclude safe dose escalation. Third-course sSRS may be best suited for oligoprogressive disease in previously irradiated sites among patients with adequate performance status and sufficient interval from prior radiation.

Preclinical studies provide important insight into the kinetics and extent of spinal cord recovery and tolerance after re-irradiation. In rodent models, Ruifrok et al. (1992) demonstrated that the ED50 for re-irradiation increases most rapidly within the first month post-treatment in 3 week old rats, with limited additional recovery thereafter; notably, even at 6 months, full recovery to baseline tolerance was not achieved [20]. However, adult rats showed more substantial recovery between 2 and 6 months, highlighting the influence of developmental stage on spinal cord repair. Knowles et al. (1983), using a guinea pig model, found that the ED50 for paralysis one year after an initial 10 Gy exposure was nearly equivalent to that of previously unirradiated animals (19.5 Gy vs. 20.5 Gy, respectively), suggesting that meaningful recovery is possible over longer intervals [21]. Ang et al. (2001) was a rhesus monkey study that demonstrated substantial recovery of spinal cord tolerance, with results showing up to 76%, 85%, and 101% of the original dose after a 1, 2, and 3-year interval, respectively. Furthermore, swine models in Medin et al. (2012) that were reirradiated with spinal radiosurgery one year after prior fractionated treatment did not exhibit a higher incidence of neurologic injury compared to those that received radiosurgery alone, suggesting no effect from prior radiation at 1 year. In light of these findings, the absence of radiation-induced myelopathy in our cohort receiving three courses of sSRS suggests that, with appropriate patient selection and sufficient time intervals between treatments, clinically meaningful recovery of spinal cord tolerance may occur, making repeat irradiation a feasible strategy in select cases.

Sahgal et al. (2012) reviewed safe spinal cord dosing and suggested the following for re-irradiation SBRT delivered in 1 to 5 fractions [22]: cumulative thecal sac EQD2_2_ D_max_ ≤ 70 Gy, re-irradiation thecal sac EQD2_2_ D_max_ ≤ 25 Gy, re-irradiation thecal sac EQD2_2_ D_max_ to cumulative EQD2_2_ D_max_ ratio ≤ 0.5, minimum time interval to re-irradiation should be at least 5 months. In our cohort, the median cumulative thecal sac EQD2_2.5_ D_max_ was 137.9 Gy (range, 97–227.5). The median third course re-irradiation sSRS thecal sac EQD2_2.5_ D_max_ was 37.15 Gy (range, 21.8–57.8). Thus, the re-irradiation sSRS thecal sac EQD2_2.5_ D_max_ to cumulative EQD2_2.5_ D_max_ ratio was 0.27, which remains below recommendation of 0.5 from Sahgal et al. (2012). Our median time interval from the second course to third course of sSRS was 12.1 months (range, 3.6–21.2), exceeding the recommended 5-month minimum. Direct numerical comparison to Sahgal’s thresholds is approximate given our use of α/β = 2.5 Gy versus their α/β = 2 Gy; however, cumulative doses exceeded absolute thresholds under either assumption, reinforcing the importance of multidisciplinary review in these complex cases.

Drawing on our experience and the existing literature, we suggest the following candidate considerations for third-course sSRS: (1) oligoprogressive disease limited to a previously irradiated spinal segment; (2) a minimum interval of at least 5 months [22] from the prior course of sSRS, with longer intervals preferred and consistent with preclinical data supporting spinal cord recovery over time [21]; (3) KPS ≥ 70; (4) cumulative thecal sac EQD2₂ Dmax that permits re-irradiation while maintaining a re-irradiation-to-cumulative dose ratio below 0.5^22^; and (5) multidisciplinary consensus that alternatives have been considered and are not feasible or preferred. These considerations are offered as a framework, not a prescription for this rare, complex patient population.

Our study has important limitations, most notably our small sample size. The small sample size reflects the rarity of this clinical scenario. A patient with spinal metastases must experience local failure after two prior courses of sSRS, maintain sufficient performance status and systemic disease control to remain a candidate for further irradiation, and survive long enough to be captured in follow-up. Only 6 patients meeting eligibility criteria were identified, underscoring the rarity of this indication. Multi-institutional collaboration will be necessary for more definitive conclusions. The study’s retrospective design may lead to underreported outcomes and formal epidural spinal cord compression (ESCC) grading was not systematically collected and could not be reported for all patients. Future studies should adopt systematic ESCC grading at each re-irradiation decision point. In addition, while the 6-month rPFS rate of 85.7% is encouraging, it must be interpreted with caution, as the median follow-up for segments without progression was only 2.8 months. Critically, the short median follow-up of 8.9 months is insufficient to exclude late radiation myelopathy or neuritis, and their absence should not be taken as definitive evidence of long-term safety. Longer follow-up and prospective study are warranted. A strength of our study was that our patients were followed closely with regular follow-up and imaging, allowing for timely monitoring of radiographic progression, VCF, and neurological changes. Further study is warranted into the clinical characteristics and outcomes of patients who receive at least three courses of sSRS.

## Conclusion

Our findings suggest that three courses of salvage stereotactic spine radiosurgery (sSRS) may be a reasonable option for patients with progressive spinal metastases. While this approach appears to be both safe and effective, further study is needed to better define long-term outcomes and toxicity risks. Importantly, because the cumulative radiation dose to adjacent neural structures can exceed thresholds established in prior studies, careful multidisciplinary evaluation is essential. Consideration of surgical management or alternative ablative modalities such as radiofrequency or cryoablation should be undertaken before proceeding with additional salvage spine SRS.

## Data Availability

The data used to support the findings of this study are available from the corresponding author upon request.

## Declarations

### Previous presentations

Abstract presented at ASTRO Annual Meeting, 2024, Walter E. Washington Convention Center, Washington, DC.
